# Acute and Chronic High-Intensity Exercise Differentially Regulate the miRNA Biogenesis Pathway in Human Skeletal Muscle

**DOI:** 10.3390/genes17060626

**Published:** 2026-05-29

**Authors:** Zeyu Wu, Eveline S. Menezes, Natalia de M. Lyra e Silva, Benjamin B. Arhen, Lucas P. R. Beaupre, Craig A. Simpson, Chris McGlory, Fernanda G. De Felice, Brendon J. Gurd

**Affiliations:** 1 School of Kinesiology and Health Studies, Queen’s University, Kingston, ON K7L 3N6, Canada19esm2@queensu.ca (E.S.M.); l.beaupre@queensu.ca (L.P.R.B.);; 2 Department of Biomedical and Molecular Sciences and Centre for Neuroscience Studies, Queen’s University, Kingston, ON K7L 2V5, Canada; 3 School of Medicine, Queen’s University, Kingston, ON K7L 3L4, Canada

**Keywords:** microRNA biogenesis, exercise, training, miR-133

## Abstract

**Background/Objectives**: MicroRNAs (miRNAs) are key regulators of skeletal muscle adaptation; however, the extent to which exercise modulates the miRNA biogenesis pathway remains poorly understood. To investigate the impact of acute and chronic high-intensity exercise on components of miRNA biogenesis, and whether such changes are reflected in miRNA expression across stages of their biogenesis, we performed secondary analyses of muscle biopsy samples from two previously published studies. **Methods**: Muscle biopsies were analyzed from the following protocols: nine men and eight women pre- and 3 h post- a bout of high-intensity interval cycling exercise (HIIE), and eleven men and eight women pre- and post- a 6-week period of high-intensity interval training (HIIT) or non-exercise control. mRNA expression of components of miRNA biogenesis including *Drosha*, *Exportin-5*, *Dicer*, and *Ago2* were assessed following HIIE using RT-qPCR and their protein abundance was measured following HIIT using Western blotting. Primary (*pri-miR-133a1*, *-133a2*, *-133b*) and mature (*miR-133a-3p*, *-133a-5p*, *-133b*) miRNA expression were quantified following HIIT. **Results**: An acute bout of HIIE significantly decreased *Drosha* mRNA (*p* < 0.05) and resulted in a reduction in *Dicer* mRNA that approached significance (*p* < 0.10). Following 6 weeks of HIIT, no significant changes were detected in the protein abundance of Drosha, Exportin-5, Dicer, or Ago2. HIIT did not alter *miR-133* expression at either the primary or mature transcript level across all isoforms. **Conclusions**: This study highlights the complexity of miRNA regulation in skeletal muscle and underscores the need for further research examining the temporal and mechanistic control of miRNA biogenesis in response to exercise.

## 1. Introduction

Endurance training activates signaling pathways that increase mRNA expression, stimulate protein synthesis, and improve mitochondrial content and aerobic capacity [1]. Recently, the dogma that exercise-induced changes in mRNA levels determine changes in protein abundance has been challenged [2], leading to increased interest in post-transcriptional mechanisms governing protein abundance [3,4,5].

MicroRNAs (miRNA) are short sequences of single-stranded non-coding RNA that contribute to post-transcriptional regulation of protein synthesis through the inhibition of translation and/or degradation of target mRNA transcripts [6,7]. miRNA biogenesis involves transcription of primary miRNA (pri-miRNA) from the genome and post-transcriptional processing of pri-miRNA to precursor (pre-miRNA) and mature miRNA [8,9]. This extensive post-transcriptional regulation of miRNA adds a layer of complexity to miRNA-mediated control in skeletal muscle and highlights two key areas of miRNA biology that require further investigation in the context of exercise: (1) regulation of the miRNA biogenesis pathway, and (2) the effects of exercise on the expression of primary/precursor and mature miRNAs.

The processing of miRNA begins within the nucleus, where, following transcription, pri-miRNA forms a stem–loop structure that is recognized and cleaved by the Drosha–DGCR8 complex. Drosha/DGCR8 generates a hairpin-shaped precursor miRNA (pre-miRNA) that is exported from the nucleus to the cytoplasm by Exportin-5. Once in the cytoplasm pre-miRNA is further processed by Dicer to become mature, single-stranded miRNAs. Mature miRNAs associate with Argonaute (Ago) proteins and are incorporated into the RNA-induced silencing complex (RISC), which binds complementary mRNA targets resulting in translational repression or mRNA degradation [8,9].

Preliminary work in human skeletal muscle suggests that components of the miRNA biogenesis pathway are regulated by exercise. For example, resistance exercise increases Dicer [10] and decreases Exportin-5 [11] protein content. Further, acute bouts of both resistance and moderate intensity endurance exercise alter the expression of *Drosha*, *Exportin-5*, and *Dicer* mRNA [10,11,12]. We are unaware of studies that have explored the effect of exercise on Ago2 mRNA or protein despite its role as the terminal effector of the miRNA biogenesis pathway and a key determinant of mature miRNA stability and activity [13,14]. At present, the impact of exercise on the expression of the miRNA biogenesis pathway remains poorly characterized, and the effects of acute and chronic high intensity aerobic exercise have yet to be studied. Thus, the primary purpose of this study was to determine the acute and chronic effects of high-intensity exercise on the expression of Drosha, Exportin-5, Dicer, and Ago2.

To date, most exercise studies in skeletal muscle have only quantified mature miRNA, failing to investigate the impact of exercise on upstream transcripts [15,16,17] limiting insight into the regulatory processes governing miRNA expression. However, recent observations suggest that regulation of miRNA expression may be under post-transcriptional control. Specifically, mature miRNA levels can be poorly correlated with corresponding pri- and pre-miRNAs [18] and mature miRNAs derived from the same primary transcript can exhibit divergent expression patterns [11,19]. Despite the potential implications for our understanding of miRNA regulation, few studies have directly compared pri- *and* mature miRNA expression following exercise. Therefore, as a secondary and exploratory aim, we examined whether training-induced changes in pri- and mature coordinate following high intensity aerobic training. Specifically we examined changes in the *miR-133* family (*miR-133a-1*, *miR-133a-2*, and *miR-133b*) an exercise responsive miRNA that constitutes a substantial proportion of skeletal muscle miRNA content [6,17,20] and is purported to influence the expression of proteins relevant to endurance adaptation, including SIRT-1, Cdc42, and IGF1R [21,22,23].

## 2. Materials and Methods

### 2.1. Study Design

The current study performed a sub-analysis using muscle biopsy samples collected from two previously published studies: an acute high-intensity interval exercise study (HIIE; [24]) and a 6-week high-intensity interval training study (HIIT; [25]). Full details regarding the experimental protocols for both studies have been published previously and are available elsewhere [24,25].

### 2.2. Participants

A total of seventeen (n = 9 males; n = 8 females) individuals volunteered to participate in the HIIE study [24] and nineteen (n = 11 males; n = 8 females) volunteered to participate in the HIIT study [25]. Participants in both studies were recruited if they were: (1) recreationally active (i.e., self-reported structured physical activity <3 h per week), (2) between 18 to 40 years of age, (3) not involved in any concurrent exercise training program, and (4) had a body mass index (BMI) less than 30 kg/m^2^. Participants were excluded from the study if they: (1) were taking any performance enhancing supplements and/or prescription medication intended for treating underlying conditions (e.g., metformin, antidepressants), (2) were current smokers, and/or (3) had a history of cardiometabolic disease (e.g., diabetes, hypertension). Physical activity levels and readiness were assessed using the Physical Activity Readiness Questionnaire (PAR-Q). Participants in both studies performed exercise on a Monark Ergomedic 874E cycle ergometer (Monark, Varberg, Sweden).

Both studies were approved by the Health Sciences Research Ethics Board at Queen’s University in accordance with the Declaration of Helsinki and all participants provided verbal and written consent prior to any data collection. Prior to participant recruitment, the Open Science Framework was used to register the HIIE (https://doi.org/10.17605/OSF.IO/U7PX9) and the HIIT (https://doi.org/10.17605/OSF.IO/7FM3N) study.

### 2.3. Experimental Design of HIIE and HIIT Study

**Figure 1:**
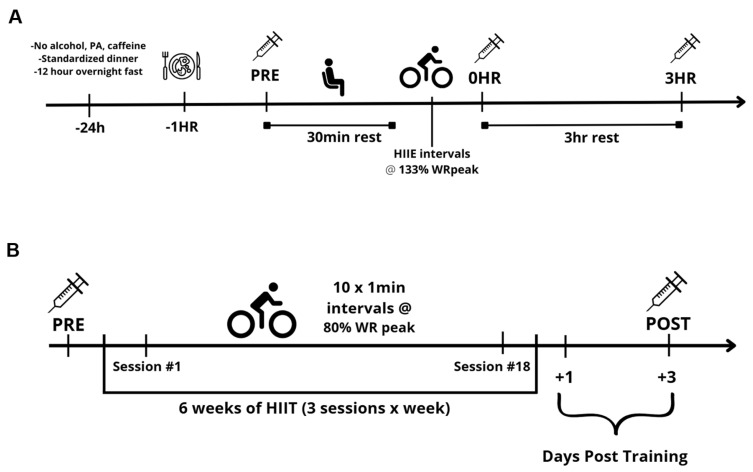
(**A**) Overview of HIIE study protocol. (**B**) Experimental timeline of HIIT study. Syringes indicate biopsy time points. Please see methods for details on pre- and post-training protocols for HIIT study.

Details regarding the HIIE crossover study are published [24] and presented in Figure 1A. In brief, participants initially underwent a VO_2peak_ test during the week prior to their first experimental trial to determine peak aerobic power (WR_peak_). After this evaluation, participants were randomized to complete one of two high-intensity interval exercise sessions at either maximal intensity (100% WR_peak_) or supramaximal intensity (133% WR_peak_). Muscle biopsies were taken from the vastus lateralis before (Pre), immediately (0 h) and 3 h (3 h) after each exercise session. The 3 h post-exercise biopsy timepoint was included to capture early transcriptional responses to exercise as PGC-1α mRNA expression, the primary outcome of our initial study [24], is expected to be elevated around this time [26]. Because our previous work demonstrated that genes typically responsive to exercise intensity were not differentially expressed between conditions or across time point [24], only samples from the supramaximal condition (133% WRpeak; Pre and 3 h) were included in the current analysis. The supramaximal trial consisted of a 5-min loadless warmup followed by six 1-min intervals of high intensity cycling at 133% of WR_peak_ interspersed by 1 min of loadless cycling.

Details regarding the HIIT parallel-arm randomized controlled trial are published [27] and presented in Figure 1B. The HIIT study consisted of a 6-week supervised training intervention (3 sessions/week) or a non-exercise control condition. Participants underwent physiological testing during the week before (Pre) and 72 h after (Post) the 6-week intervention. Each session began with a five-minute warm-up without any load, followed by ten 1-min intervals of high-intensity cycling at workload corresponding to 80% of peak aerobic power, with each interval separated by one minute of load-less cycling [28]. The workload was calculated from each participant’s preliminary exercise test and held constant throughout the 6-week intervention. Participants in the control group were instructed to maintain their usual levels of physical activity throughout the study period.

### 2.4. Muscle Biopsies

All muscle biopsy samples were obtained in our laboratory as previously described [29,30]. Briefly, muscle biopsies were taken from the lateral side of the vastus lateralis under superficial local anesthesia (2% lidocaine with epinephrine) using a Bergstrom needle biopsy technique [31] with manual suction. For both studies, muscle biopsy samples were obtained from a single leg during each experimental trial and immediately frozen in liquid nitrogen and stored at −80 °C until analysis. Muscle samples from the HIIT study were used for western blot and miRNA expression analysis, whereas samples from the HIIE study were used for gene expression analysis. We chose to only analyze mRNA expression in the HIIE study because previous work demonstrated that components of the miRNA biogenesis pathway exhibit transient changes in mRNA expression during the early post-exercise recovery period, however, corresponding changes in protein abundance are generally not observed within the same timeframe [10,11].

### 2.5. Western Blot Analysis

**Figure 2:**
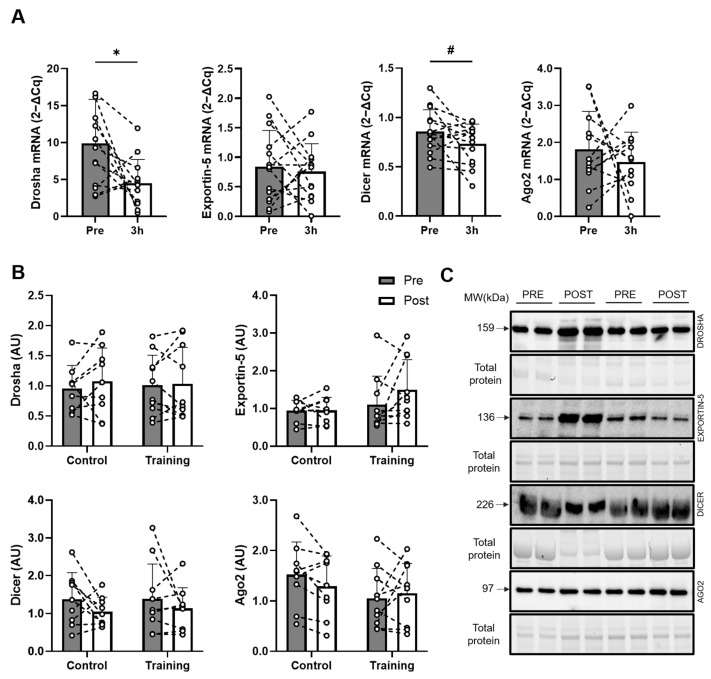
Effect of acute and chronic HIIE on miRNA biogenesis pathway. (**A**) Changes in *Drosha*, *Exportin-5*, *Dicer*, and *Ago2* mRNA expression in the *vastus lateralis* before (Pre; grey boxes) and after (3 h; white boxes) HIIE. (**B**) Impact of HIIT on miRNA biogenesis proteins. Pre (grey bar) and Post (white bar); AU, arbitrary units. (**C**) Representative western blots from two participants. Note: Symbols denote significant (* *p* < 0.05) and approaching significant (# *p* < 0.10) differences versus Pre.

To investigate whether transient changes in the mRNA expression of the miRNA biogenesis pathway accumulate into corresponding changes in protein abundance following repeated exercise bouts, Western blot analysis was performed on the HIIT study samples to determine the protein abundance of Drosha, Exportin-5, Dicer, and Ago2 before and after HIIT training (HIIE samples were not analyzed due to insufficient quantity of muscle sample). Briefly, ~20 mg of muscle tissue was homogenized, and protein concentrations were quantified by protein assay (BCA Protein Assay Kit 23225, Pierce, Rockford, IL, USA). The homogenized tissue was then diluted in 4X Laemmli buffer and deionized water). Fifteen micrograms of total protein was loaded in different wells on 4–15% precast Criterion TGX Stain-Free gels (Bio-Rad, Hercules, CA, USA). A stain-free system (Bio-Rad, Hercules, CA, USA) was used as a loading control, with protein expression normalized to total protein loaded per lane (see Figure 2C for representative blots). Muscle lysates were separated via gel electrophoresis (45 min @ 200 V) and transferred to polyvinylidene difluoride (PVDF) membranes (7 min @ 25 V). Membranes were blocked in 5% bovine serum albumin (BSA) for 90 min and incubated overnight at 4 °C with primary antibodies (Abcam, Waltham, MA USA) against Drosha (ab12286), Exportin-5 (ab129006), Dicer (ab315232), and Ago2 (ab32381) in TBST with 5% BSA. Antibodies were used at a 1:1000 dilution except for Drosha and Dicer, which were used at 1:2000, and 1:500, respectively. The following day, membranes were incubated for 60 min at room temperature with goat anti-rabbit horseradish peroxidase (HRP) conjugated secondary antibodies (ThermoFisher Scientific, Waltham, MA, USA, no. 7074S) at a dilution of 1:1000 before being exposed to a chemiluminescence solution (Clarity™ Western ECL Substrate [Bio-Rad, Hercules, CA, USA]). Images were taken with a Chemidoc Imaging system fitted (Bio-Rad, Hercules, CA, USA) and densitometry was performed with Image Lab 6.1 software (Bio-Rad, Hercules, CA, USA).

### 2.6. RNA Extraction

RNA extraction was performed using two separate protocols for the HIIE and HIIT study. In the HIIE study, RNA extraction was performed as previously described [32] by grounding frozen muscle samples into a fine powder using a mortar and pestle before suspension in a buffer containing guanidine thiocyanate, sodium citrate, sarkosyl, and β-mercaptoethanol [33,34]. Extracted RNA samples from the HIIE study had an average 260/280 ratio of 2.00 ± 0.04 (mean ± standard deviation [SD]), indicating high purity. In the HIIT study, a different protocol was used to enable efficient co-isolation of miRNAs, with RNA extracted from 25 mg of muscle tissue using the AllPrep DNA/RNA/miRNA Universal Kit (Qiagen, Mississauga, ON, Canada). Extracted RNA samples from the HIIT study had an average 260/280 ratio of 1.95 ± 0.02.

### 2.7. RT-qPCR

In the HIIE study, one microgram (1 μg) of RNA was reverse transcribed using the QuantiTect Reverse Transcription Kit (Qiagen, Mississauga, ON, Canada) and mRNA levels were determined using a QuantStudio™ 3 Real-Time PCR System (Thermo Fisher Scientific, Waltham, MA, USA). Forward and reverse primer sequences were designed using Primer-BLAST (NCBI) and synthesized by Integrated DNA Technologies (IDT, Coralville, IA, USA). All samples were run in duplicate reactions containing 50 ng of complementary DNA (cDNA) and GoTaq PCR Master Mix containing SYBR Green (Promega, Madison, WI, USA). Results were analyzed according to the ΔCq method, using TATA-binding protein (*TBP*) as the housekeeping gene. Primer sequences are provided within Table 1.

### 2.8. miRNA RT-qPCR

In the HIIT study, 500 nanograms (500 ng) of input RNA was used for cDNA synthesis using High-Capacity RNA-to-cDNA kit (Thermo Scientific) to quantify the expression of primary miRNAs (*pri-miR-133a1*, *pri-miR-133a2*, and *pri-mirR-133b*; see Table 2). An additional 10 ng of input RNA was required for cDNA synthesis using TaqMan Advanced miRNA cDNA Synthesis Kit (Thermo Scientific) for analysis of mature miRNAs (*miR-133a3p*, *miR-133a5p*, and *miR-133b*; see Table 2). qPCR was also performed on a Quantstudio 3 Real-Time PCR System (Thermo Scientific) but with TaqMan Master Mix (Thermo Scientific). Primary miRNAs were normalized to GAPDH while mature miRNAs were normalized to mean of three housekeeping miRNAs (*miR-191*, *miR-361*, and *miR-320a*). Relative fold changes were determined using the 2^−ΔΔCT^ method [35].

### 2.9. Statistical Analysis

Statistical analyses were performed using SPSS Statistics version 29 (IBM Corp., Armonk, NY, USA). All data are presented as mean ± standard deviation (SD). Statistical significance was set at *p* < 0.05 and trends approaching statistical significance were considered at *p* < 0.10.

Independent *t*-tests were used to compare male and female participants’ characteristics from both studies (see Table 3). Gene expression analyses were performed using 2^−ΔCq^ method for the HIIE study and 2^−ΔΔCT^ method for the HIIT study. We calculated ΔΔCt by subtracting the mean Pre ΔCt value of all participants from each individual sample’s ΔCt value. For the HIIE study, changes in gene expression over time (Pre and 3 h) were analyzed using paired *t*-tests to examine the effects of a single bout of high-intensity interval exercise (133% WR_peak_) on *Drosha*, *Exportin-5*, *Dicer*, and *Ago2*. For the HIIT study, differences in protein abundance and miRNA expression between groups (exercise and control) and over time (Pre and Post) were evaluated using a two-way mixed-design ANOVA, with group as the between-subjects factor and time as the within-subjects factor. Significant main effects and/or interactions were subsequently investigated using Bonferroni’s post hoc tests.

Prior to formal analysis, the Shapiro-Wilk test and Levene’s test were performed to ensure the data met assumptions of normality and homogeneity of variance. A log transformation was applied in cases where the data did not meet these assumptions [36]. All outliers were identified using Tukey’s boxplots (see  Supplementary Figures) and removed prior to analysis.

Effect sizes were calculated to provide additional context to the statistical analyses and to quantify the magnitude of observed effects. Effect sizes were calculated using G*Power 3.1. For independent *t*-tests and paired *t*-test, Cohen’s d was computed [37] and values were interpreted using conventional thresholds: 0.2 (small effect), 0.5 (medium effect), and 0.8 (large effect). For the ANOVA analyses in the HIIT study, effect sizes were assessed using partial eta squared (η^2^) and interpreted based on the following benchmarks: 0.01 (small effect), 0.06 (medium effect), and 0.14 (large effect) [37]. By reporting effect sizes alongside *p*-values, we aim to provide a more comprehensive understanding of the practical significance of our findings beyond mere statistical significance. Since no formal a priori power calculation was conducted for the outcomes examined in the present study, sensitivity analyses were performed to determine the minimum detectable effect size for each main outcome based on the available sample size, α = 0.05, and power = 0.80 (See Supplemental Tables S1–S4).

## 3. Results

Participant characteristics for both studies are presented in Table 3. Body mass (*p* < 0.001; d = 2.19), height (*p* < 0.001; d = 1.95), and BMI (*p* = 0.006; d = 1.33), in the HIIE study were statistically different between male and female participants. In the HIIT study, body mass (*p* = 0.001; d = 1.72), height (*p* = 0.009; d = 1.43), and WR_peak_ (*p* < 0.001; d = 2.48) were statistically different between male and female participants. There were no differences (*p* > 0.4) in participant characteristics between training and control group in the HIIT study (Table 4). Effect sizes and final sample sizes for all analyses are included in Supplemental Tables S1–S4.

Changes in *Drosha*, *Exportin-5*, *Dicer*, and *Ago2* mRNA expression 3 h after an HIIE session are presented in Figure 2A. *Drosha* (*p* = 0.02; d = 0.77) expression decreased after HIIE while a decrease in *Dicer* (*p* = 0.08; d = 0.50) expression approached significance. No significant changes were observed in the expression of *Exportin-5* (*p* = 0.67; d = 0.12) or *Ago2* (*p* = 0.47; d = 0.27). Changes in skeletal muscle protein expression of Drosha, Exportin-5, Dicer, and Ago2 after 6 weeks of HIIT are presented in Figure 2B. No significant main effects of time (all *p* > 0.13; ƞ^2^ < 0.13), condition (all *p* > 0.16; ƞ^2^ < 0.11), or interaction effects (all *p* > 0.17; ƞ^2^ < 0.11) were observed for any of the proteins probed.

**Figure 3:**
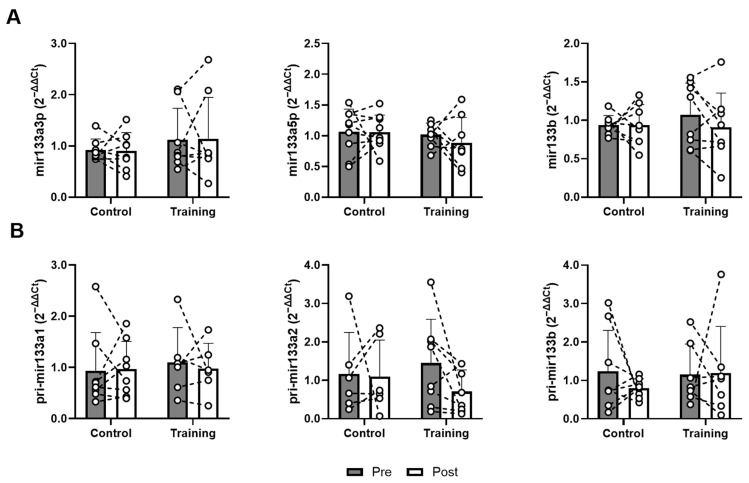
Effect of chronic HIIT on *miR-133* expression. (**A**) Impact of HIIT on mature miRNAs (*miR-133a3p*, *-133a5p*, and *133b*). (**B**) Impact of HIIT on primary miRNAs (*pri-miR133a1*, *-133a2*, and *133b*). Pre (grey bar) and Post (white bar).

Changes in miRNA expression following 6 weeks of HIIT is also shown in Figure 3. No significant main effects of time (all *p* > 0.35; ƞ^2^ < 0.06) or interaction effects (all *p* > 0.35; ƞ^2^ < 0.06) were observed for any of the mature miRNAs probed (Figure 3A). No significant main effects of time (all *p* > 0.27; ƞ^2^ < 0.10) or interaction effects (all *p* > 0.36; ƞ^2^ < 0.07) were observed for any of the pri-miRNAs probed (Figure 3B).

## 4. Discussion

The aim of the present study was to characterize the expression of the miRNA biogenesis pathway following acute and chronic high-intensity interval exercise and examine how training modulates primary and mature *miR-133*. The primary findings of this study are: (1) an acute bout of HIIE decreases the mRNA expression of *Drosha* and *Dicer*, (2) 6 weeks of HIIT had no effect on the protein content of components of the miRNA biogenesis pathway, and (3) HIIT did not alter *miR-133* expression at either the primary or mature transcript level across all isoforms.

Previously, an acute bout of endurance exercise (60 min at 70% VO_2max_) was observed to significantly upregulate mRNA expression levels of *Drosha*, *Dicer*, and *Exportin-5* [12], with similar responses observed following even shorter durations and at lower exercise intensities (45 min at 55% VO_2max_) [38]. To the best of our knowledge, the current study is the first to report decreased expression of *Drosha* and *Dicer* mRNA (Figure 2A) following a single bout of aerobic exercise. Given that the present study shares the same 3 h post-exercise sampling time point as previously mentioned studies [12], temporal differences in gene expression are unlikely to fully explain these divergent findings. Rather, the decrease in *Drosha* and *Dicer* mRNA observed here may be attributed to differences in prescribed exercise intensity. High intensity exercise–133% of WR_peak_ in the current study–elicits a unique cellular environment characterized by pronounced metabolic/mechanical stress which may influence transcriptional regulation or stability of *Drosha* and *Dicer* mRNAs [39,40]. For instance, c-Myc—a transcriptional activator of *Drosha* [41]—is downregulated following high intensity exercise [42,43]. Similarly, *Dicer* mRNA expression is repressed by FOXO [44], a transcription factor that is activated by high cellular stress and energy demand [45]. The relationships between c-Myc and *Drosha*, and FOXO and *Dicer* suggest that high intensity exercise may produce a *Drosha*/*Dicer* response that contrasts with lower intensity aerobic exercise, a hypothesis that requires direct testing in future studies.

To date, no studies have examined the effects of chronic high-intensity interval training on the protein content of the miRNA biogenesis pathway. We found that 6 weeks of HIIT did not alter the protein abundance of key miRNA processing machinery (Figure 2B). These findings appear to align with previous work reporting no significant changes in Drosha, Exportin-5, or Dicer mRNA levels following 8 days of aerobic exercise [12], suggesting that training does not induce measurable adaptations in factors central to miRNA maturation and transport. Given that exercise training has been shown to alter the expression of several miRNAs [6,12,17], the lack of change in miRNA processing protein abundance observed here suggests that training-induced modulation of miRNA levels likely occurs independent of the core biogenesis machinery [46]. Rather, regulation may involve transcriptional control of pri-miRNAs, shifts in processing efficiency, or altered stability and turnover of mature miRNAs.

Among these possible mechanisms, regulation of mature miRNA stability is particularly compelling given the central role of Ago proteins. While Ago does not play a direct role in miRNA biogenesis, mature miRNAs must be loaded onto Ago to form the miRNA-induced silencing complex (miRISC) which ultimately mediates target recognition and gene silencing [47,48,49]. Indeed, accumulating evidence further indicates that Ago contributes to the post-transcriptional regulation of miRNA expression, as Ago2 binding protects mature miRNAs from exonuclease-mediated decay [50]—a concept supported by studies reporting that global miRNA levels are closely linked to Ago2 abundance/availability [14,27,51]. To our knowledge, this study is the first to characterize the expression of Ago2 in response to exercise. We observed no changes in Ago2 mRNA/protein following HIIE and HIIT—an intriguing result given that Ago2 is highly regulated by post-translational modifications and protein-ligand interactions [52,53,54]. While our results suggest that exercise may not regulate Ago2, more research is required to verify our findings.

The present study also focused on *miR-133*, an exercise responsive miRNA that is implicated in muscle remodeling and mitochondrial adaptation [6,17,21]. It is worth noting that previous exercise studies examining *miR-133* have typically assessed only a single isoform [17,20] or have not specified the isoform measured [16,55], limiting interpretability, as miRNA isoforms can exhibit distinct expression profiles [11,23] despite sharing the same target repertoire. Moreover, research on primary miRNA expression following exercise is virtually non-existent, a critical knowledge gap considering the growing evidence showing discordant regulation between mature miRNAs and their upstream constituents [11,18]. We believe this study is the first to characterize all *miR-133* isoforms at both the primary and mature stages of miRNA biogenesis. No significant differences in *miR-133* isoform expression were observed at either the primary or mature transcript level following HIIT, suggesting that training did not alter *miR-133* regulation across stages of miRNA biogenesis. Current analyses of *miR-133* response to aerobic exercise appear to vary, with some reporting decreases after prolonged training [16,17] or finding no persistent changes [12]. One possible explanation for the inconsistency in *miR-133* expression could be attributed to differences in initial training status. Notably, studies that appear to show a decrease in *miR-133* expression involve more fit participants [23] whereas studies reporting minimal change tended to recruit untrained or recreationally active participant [12]. Alternatively, it is also possible that the duration of our intervention was not sufficient to elicit detectable changes in mature *miR-133*, as studies showing downregulation [17] typically prescribed longer training periods (e.g., ≥12 weeks). Taken together, these findings indicate that mature *miR-133* expression does not follow a uniform pattern in response to aerobic exercise but is shaped by differences in participant training status and the duration over which the exercise stimulus is applied.

## 5. Limitations and Future Directions

The present study has several limitations that should be considered when interpreting these findings. This work represents a secondary analysis of two previously conducted studies, neither of which were powered to detect changes in the outcomes examined here. As a result, some non-significant findings may reflect insufficient statistical power, particularly for outcomes in which outlier removal further reduced the number of observations available for analysis. Furthermore, we cannot *fully* characterize the response of the miRNA biogenesis pathway or determine how these responses relate to changes in *miR-133* expression due to the limited temporal resolution of our current analyses. Previous work has shown that the mRNA expression of components of the miRNA biogenesis pathway can remain altered up to 24 h after an acute bout of resistance exercise [10]. Therefore, it is possible that the decreases in *Drosha* and *Dicer* mRNA observed 3 h post HIIE persisted beyond this time point, resolved later in recovery, or preceded a delayed increase in expression. Similarly, the protein abundance of the miRNA biogenesis pathway may have changed at earlier time points during the 6-week HIIT intervention. However, because training adaptations likely reduced the physiological disturbance imposed by the same absolute exercise stimulus, transient changes in protein abundance may have been attenuated by the post-training biopsy. Future studies should include multiple post-exercise and mid-intervention sampling time points, and be adequately powered, to determine whether the lack of observed change reflects reduced exercise disturbance, and/or adaptation/habituation to the repeated exercise stimulus.

More broadly, additional research is needed to clarify the mechanisms that regulate miRNA expression in skeletal muscle, particularly in response to exercise. While the pre-sent findings suggest that high-intensity exercise may modulate select components of the miRNA biogenesis pathway, the downstream consequences for mature miRNA expression and function remain unclear. Future work should integrate measurements of primary, precursor, and mature miRNAs with assessments of miRNA biogenesis pathway. Importantly, these responses should be examined across a broader range of exercise prescriptions (e.g., training duration, intensities, modalities) with consideration of how these responses may be modified by the populations studied (e.g., training status, age, sex, and health status).

## 6. Conclusions

The current study explored the effects of HIIE and HIIT on the expression of the miRNA biogenesis pathway and the regulation of *miR-133*. Our results demonstrated that *Dicer* and *Drosha* mRNA significantly decreased following a bout of HIIE at 133% of WR_peak_. Despite seeing acute response of the miRNA biogenesis pathway, no changes in protein abundance were observed following 6 weeks of training. Consistent with these findings, HIIT did not alter *miR-133* expression at either the primary or mature transcript level across all isoforms. At present, exercise-mediated control of the miRNA biogenesis pathway remains poorly understood. Few studies have systematically investigated the components of this pathway in response to exercise, and those that have lack longitudinal designs or detailed investigations at both the transcriptional and protein levels. Our study provides a crucial starting point for understanding the regulation of miRNA biogenesis machinery in skeletal muscle in response to high-intensity exercise, offering insight into how these processes may (or may not) translate to changes in miRNA expression. Future studies should investigate how the miRNA biogenesis pathway responds to varying exercise intensities and modalities with a particular focus on its temporal dynamics, regulatory network, and impact on miRNA expression.

## Figures and Tables

**Table 1:** mRNA forward and reverse sequences of analyzed genes.

Genes	Forward Sequence	Reverse Sequence
*Drosha*	ACAACGATCCAGGCCAGATGA	AGGACGACAGGGCTTGATGG
*Exportin-5*	GCCCTTCCATGCAGCAAGAC	GGCTTTACAAAGACACGAAGCA
*Dicer*	ACACTGGCTCAGGGAAGACA	AGCAACCTGGTTTGCAGAGT
*Ago2*	CAAGAACGAGCGGGTTGGGA	TTGTCGTCCCAGAGGACGTG
*TBP*	AGACGAGTTCCAGCGCAAGG	GCGTAAGGTGGCAGGCTGTT

**Table 2:** Classification and identification number of Taqman probes.

Genes	ID Number
*Pri-miR-133a-1*	Hs03303117_pri
*Pri-miR-133a-2*	Hs03303121_pri
*Pri-miR-133b*	Hs03303651_pri
*miR-133a-3p*	478511_mir
*miR-133a-5p*	478706_mir
*miR-133b*	480871_mir
*miR-361-5p*	478056_mir
*miR-191-5p*	477952_mir
*miR-320-3p*	478594_mir
*GAPDH*	Hs99999905_m1

**Table 3:** Participant baseline characteristics for HIIE and HIIT study.

	Age (yr.)		Body Mass (kg)		Height (cm)		BMI (kg/m^2^)		VO_2peak_ (mL/min/kg)		WR_peak_ (Watts)	
HIIE Study												
Males (n = 9)	22.4	± 4.6	84.0	± 9.8 *	180.3	± 4.6 *	26.1	± 3.0 *	43.5	± 7.4 *	251.9	± 25.5 *
Females (n = 8)	21.6	± 2.3	61.2	± 11.2	167.2	± 8.1	21.9	± 3.3	36.3	± 6.4	182.0	± 44.2
Total (n = 17)	22.2	± 3.5	73.3	± 15.5	173.8	± 9.5	24.1	± 3.4	39.9	± 7.7	227.3	± 43.6
HIIT Study												
Males (n = 11)	23.9	± 6.2	76.2	± 6.8 *	175.2	± 6.6 *	24.8	± 2.2			250.8	± 31.3 *
Females (n = 8)	23.1	± 6.2	61.4	± 9.2	167.0	± 3.8	22.1	± 3.7			176.7	± 25.0
Total (n = 19)	23.6	± 6.2	70.0	± 10.7	171.7	± 6.9	23.7	± 3.2			217.9	± 47.3

Values presented as mean ± SD. * Significant difference (*p* < 0.05) between male and female cohorts. BMI, body mass index, VO_2peak_, peak oxygen uptake, WR_peak_, peak work rate.

**Table 4:** Participant characteristics of HIIT study organized by conditions including before and after training WRmax data.

	Exercise Group (n = 10; 5M/5F)		Control Group (n = 9; 6M/3F)	
Age (yr.)	22.8	± 4.6	24.4	± 8.3
Height (cm)	171.1	± 6.8	172.4	± 7.8
BMI (kg/m^2^)	24.2	± 3.2	23.0	± 3.6
WRmax (Watts)				
Pre	211.8	± 58.7	228.3	± 33.3
Post (24 h)	223.9	± 63.1	221.3	± 47.7
Post (72 h)	227.0	± 64.7	231.5	± 44.9

Values presented as mean ± SD.

## Data Availability

Data generated or analyzed during this study are available from the corresponding author upon reasonable request. Registration of this project can be found on the Open Science Framework for the HIIE (https://doi.org/10.17605/OSF.IO/U7PX9) and the HIIT (https://doi.org/10.17605/OSF.IO/7FM3N) study.

## Supplementary Materials

The following supporting information can be downloaded at: https://www.mdpi.com/article/10.3390/genes17060626/s1, Figure S1: Distribution of miRNA biogenesis pathway expression values in HIIE study used for outlier identification; Figure S2: Distribution miR-133 expression values in HIIT study used for outlier identification; Table S1: Effects of HIIE on the mRNA expression of the miRNA biogenesis pathway, Table S2: Effects of HIIT on the protein abundance of the miRNA biogenesis pathway, Table S3: Effects of HIIT on *miR-133* expression, Table S4: Effects of HIIT on *pri-miR-133* expression.

## Abbreviations

The following abbreviations are used in this manuscript:

miRNA	MicroRNA
HIIE	High-Intensity Interval Exercise
HIIT	High-Intensity Interval Training
pri-miRNA	Primary MicroRNA
pre-miRNA	Precursor MicroRNA
PAR-Q	Physical Activity Readiness Questionnaire
VO_2_peak	Peak Oxygen Uptake
WR_peak_	Peak Work Rate
RT-qPCR	Reverse Transcription Quantitative PCR
FOXO	Forkhead Box O
c-Myc	Cellular Myc Oncogene
