# Supplementary material for: Acute and Chronic High-Intensity Exercise Differentially Regulate the miRNA Biogenesis Pathway in Human Skeletal Muscle
Source: Genes (Basel). 2026 May 29;17(6):626. doi: 10.3390/genes17060626 (PMC13298448; doi:10.3390/genes17060626)
Supplement: Supplementary file 1 [file genes-17-00626-s001.zip › genes-4296519-supplementary.pdf]

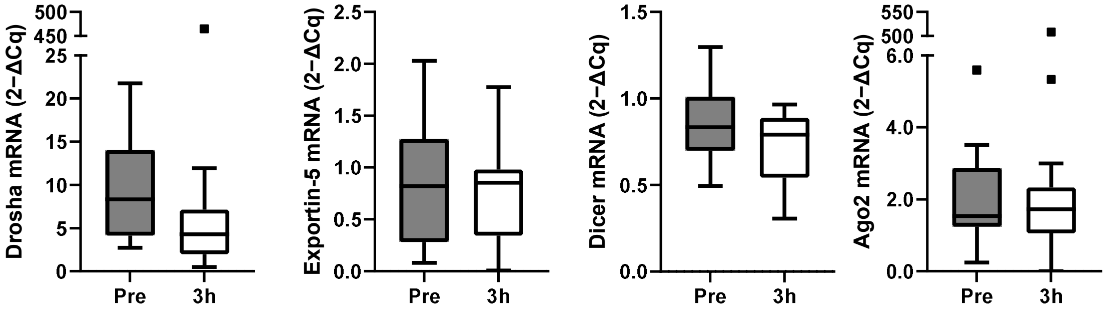

Supplementary Figure S1. Distribution of miRNA biogenesis pathway expression values in HIIE study used for outlier identification. Box-and-whisker plots represent fold change relative to baseline for *Drosha, Expotin-5, Dicer,* and *Ago2* mRNA. Outliers are represented as black squared and were identified using Tukey's boxplot method (1.5× IQR) and excluded from subsequent analyses.

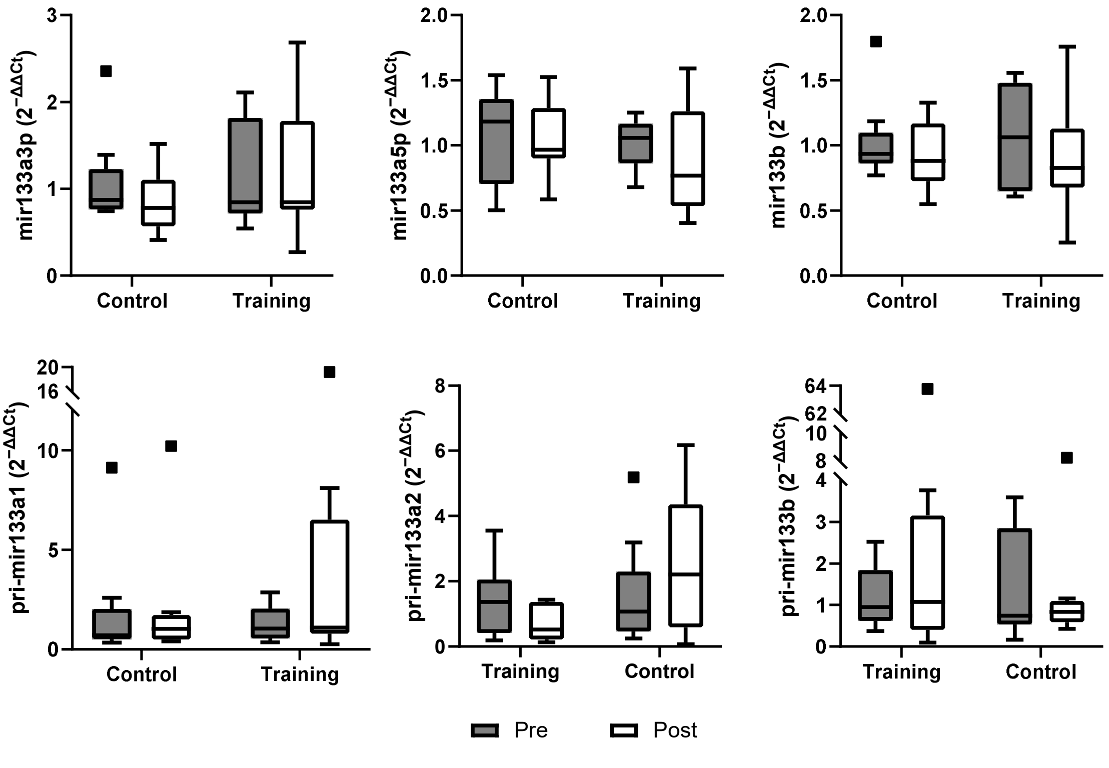

Supplementary Figure S2. Distribution *miR-133* expression values in HIIT study used for outlier identification. Box-and-whisker plots represent fold change relative to pre-training for mature miRNAs (*miR-133a-3p, miR-133a-5p,* and *miR-133b*; top panels) and primary miRNAs (*pri-miR-133a1, pri-miR-133a2,* and *pri-miR-133b*; bottom panels) in control and training groups. Outliers are represented as black squared and were identified using Tukey's boxplot method (1.5× IQR) and excluded from subsequent analyses.

Supplementary Table S1. Effects of HIIE on the mRNA expression of the miRNA biogenesis pathway

Gene	*p* value	Effect size (d)	Sample size	Minimum detectable effect size (d)
Drosha	0.02*	0.77	14 (13)	0.85
Exportin-5	0.67	0.12	14 (14)	0.81
Dicer	0.08#	0.50	14 (14)	0.81
Ago2	0.47	0.21	14 (12)	0.89

Total sample size, with sample size after outlier analysis shown in parentheses. *p* value, effect sizes (Cohen's d).
*Significant difference ($p<0.05$). # Approaching significant difference ($p<0.10$).

Supplementary Table S2. Effects of HIIT on the protein abundance of the miRNA biogenesis pathway

Protein	Interaction	Time	Condition	Sample size	Minimum detectable interaction effect size (η^2)
Drosha	p=0.56<br>η^2=0.02	p=0.42<br>η^2=0.03	p=0.98<br>η^2<0.01	19 (19)	0.13
Exportin-5	p=0.19<br>η^2=0.10	p=0.17<br>η^2=0.11	p=0.16<br>η^2=0.11	19 (19)	0.11
Dicer	p=0.82<br>η^2<0.01	p=0.13<br>η^2=0.13	p=0.85<br>η^2=0.01	19 (19)	0.14
Ago2	p=0.17<br>η^2=0.11	p=0.59<br>η^2=0.02	p=0.25<br>η^2=0.08	19 (19)	0.07

Total sample size, with sample size after outlier analysis shown in parentheses. *p* value, effect sizes (partial η^2).

Supplementary Table S3. Effects of HIIT on *miR-133* expression

miRNA	Interaction	Time	Condition	Sample size	Minimum detectable interaction effect size (η^2)
miR-133a-3p	p=0.90<br>η^2<0.01	p=0.99<br>η^2<0.01	p=0.35<br>η^2=0.06	17 (16)	0.13
miR-133a-5p	p=0.58<br>η^2=0.02	p=0.53<br>η^2=0.03	p=0.35<br>η^2=0.06	17 (17)	0.20
miR-133b	p=0.35<br>η^2=0.06	p=0.35<br>η^2=0.06	p=0.71<br>η^2<0.01	17 (16)	0.13

Total sample size, with sample size after outlier analysis shown in parentheses. *p* value, effect sizes (partial η^2).

Supplementary Table S4. Effects of HIIT on *pri-miR-133* expression

miRNA	Interaction	Time	Condition	Sample size	Minimum detectable interaction effect size (η^2)
pri-miR-133a1	*p*=0.68<br>$\eta^2<0.01$	*p*=0.82<br>$\eta^2<0.01$	*p*=0.77<br>$\eta^2<0.01$	17 (14)	0.17
pri-miR-133a2	*p*=0.36<br>$\eta^2=0.07$	*p*=0.27<br>$\eta^2=0.10$	*p*=0.89<br>$\eta^2<0.01$	17 (14)	0.24
pri-miR-133b	*p*=0.48<br>$\eta^2=0.02$	*p*=0.54<br>$\eta^2<0.01$	*p*=0.64<br>$\eta^2<0.01$	17 (15)	0.27

Total sample size, with sample size after outlier analysis shown in parentheses. *p* value, effect sizes (partial η^2).
